# Factors Associated with the Anamnestic Immune Response Following Hepatitis B Booster Vaccination in the Elderly

**DOI:** 10.3390/vaccines14020111

**Published:** 2026-01-23

**Authors:** Chen Wang, Yan Zou, Xiaofei Wang, Na Liu

**Affiliations:** 1 Zhangjiagang Center for Disease Control and Prevention, Zhangjiagang 215600, China; wangczjg@163.com (C.W.);; 2 Suzhou Center for Disease Control and Prevention, Suzhou 215100, China

**Keywords:** elderly people, hepatitis B vaccine, anamnestic immune response, hepatitis B surface antibody, influence factor

## Abstract

**Objective:** To investigate factors influencing the anamnestic immune response 9 years after hepatitis B vaccination in elderly people (aged > 60 years). **Methods:** We quantitatively tested 630 elderly people who participated in the free hepatitis B vaccination program for adults in Zhangjiagang City during 2015 for hepatitis B surface antibody (anti-HBs) titers. Three booster doses of hepatitis B vaccine were given to subjects with anti-HBs titers below 10 mIU/mL, while a single booster dose was administered to those with titers between 10 and 100 mIU/mL, in accordance with their antibody titer measurements. The post-booster anti-HBs titers were evaluated at 2–3 months. A logistic regression model was used to identify factors influencing the anamnestic immune response, and a receiver operating characteristic curve analysis was conducted. **Results:** Among the 90 participants who received three doses and the 101 participants who received one dose, baseline characteristics did not differ significantly between the two cohorts. Both groups exhibited robust anamnestic immune responses. Significant differences were observed before and after booster vaccination within each group (*Z* = −8.24, *p* < 0.001; *Z* = −8.73, *p* < 0.001). Multivariate logistic regression indicated that individuals with higher pre-booster anti-HBs titers were less likely to show weak anamnestic responses compared to those with lower pre-booster titers (*OR* = 0.30, 95% *CI*: 0.16–0.58). Furthermore, a high anamnestic immune response (>1000 mIU/mL) was significantly more frequent among subjects with pre-booster titers ≥ 4.58 mIU/mL. **Conclusions:** Booster immunization administered nine years after hepatitis B vaccination induces robust anamnestic immunity, with its magnitude significantly correlated with pre-booster anti-HBs titers. Particular attention should be given to individuals with extremely low pre-booster anti-HBs levels.

## 1. Introduction

Hepatitis B is a viral infection caused by the hepatitis B virus (HBV) and represents a major threat to global public health. HBV infection can lead to serious and long-term adverse consequences, such as liver cirrhosis and liver cancer [1,2,3]. The World Health Organization (WHO) reported in 2025 that about 1.2 million new hepatitis B infections occur each year [4]. The prevalence of HBV infection in China was 6.89%, and there are about 84 million hepatitis B surface antigen (HBsAg) carriers [5]. The burden of chronic hepatitis B carriers is the largest in the world [6,7]. Therefore, preventing hepatitis B virus infection is essential. Affordable, safe, and effective hepatitis B vaccines are the optimal tools for controlling hepatitis B transmission. Indeed, hepatitis B vaccination significantly reduced infection rates, thereby leading to a great reduction in the disease burden [8].

Individuals with a history of hepatitis B vaccination should receive booster dose(s) if they are found to have inadequate titers of hepatitis B surface antibody (anti-HBs). Most of the current studies use the anti-HBs titers of 10 mIU/mL as the reference standard, analyzing differences between individuals with anti-HBs titers ≥ 10 mIU/mL (positive) and those with titers < 10 mIU/mL (negative). However, studies on differences within the responders (anti-HB ≥ 10 mIU/mL) have rarely been reported. Among responders, individuals with higher anti-HBs titers had a lower proportion of breakthrough HBV infections compared to those with lower anti-HBs titers [9,10]. Therefore, by comparing subgroups within the population that have responded to a hepatitis B vaccine booster, the study of high immune responders’ characteristics is valuable for guiding the implementation of booster vaccination.

Considering that the efficacy of the hepatitis B vaccine is generally lower in the elderly than in the young [11,12,13], studying booster immunization in the elderly becomes more necessary. The protective efficacy of the hepatitis B vaccine is encompassed by both the concentration of anti-HBs and the magnitude of the anamnestic immune response [14,15]. The strength of the immune memory response was generally assessed in most studies by measuring the effectiveness of a booster dose [16,17,18]. In light of the pursuit of better protective efficacy, this study employed a post-booster anti-HBs titer of 1000 mIU/mL as the reference threshold for a high immune response [19]. Elderly participants were subsequently stratified according to this criterion to explore the factors influencing the anamnestic immune response 9 years after completing the hepatitis B vaccination course. Current research on immune recall responses rarely involves elderly populations. By focusing on older adults, this study can provide a reference for future research.

Furthermore, given that numerous studies on hepatitis B vaccine efficacy have incorporated chronic disease history into their analyses [20,21], the immunogenic response to vaccination in individuals with chronic diseases has become a hot topic that requires urgent investigation and resolution. This study focuses on the booster vaccine response in the elderly with chronic diseases, which contributes to the formulation of targeted booster vaccination policies for the elderly.

## 2. Materials and Methods

### 2.1. Participants

The study subjects were selected from 17,178 individuals with vaccination records from a free hepatitis B vaccination program for adults in Zhangjiagang City during 2015, in which yeast-derived hepatitis B vaccines were used. Among them, 630 participants who were older than 60 years and had completed the full course of hepatitis B vaccination were recruited, and quantitative detection of five hepatitis B serological markers was performed for these subjects. Testing revealed 167 subjects who were negative for all three markers: hepatitis B surface antigen (HBsAg), hepatitis B surface antibody (anti-HBs), and hepatitis B core antibody (anti-HBc), with anti-HBs concentrations below 10 mIU/mL. Additionally, there were 132 subjects who tested negative for HBsAg, hepatitis B e antigen (HBeAg), hepatitis B e antibody (anti-HBe), and anti-HBc, but had anti-HBs concentrations between 10 mIU/mL and 100 mIU/mL. Two cohorts were established from the above-mentioned population groups. The Ethics Committee of the Zhangjiagang Center for Disease Control and Prevention reviewed and approved this study (Approval No. 2024-01). All participants provided written informed consent.

### 2.2. Study Design

The aforementioned cohort was contacted by phone and asked to visit their local vaccination clinics for hepatitis B booster shots. To explore an optimized hepatitis B vaccine booster strategy and allocate medical resources more efficiently, this study employed a titer-stratified vaccination protocol. Participants seronegative for HBsAg, anti-HBs, and anti-HBc with anti-HBs titers below 10 mIU/mL received the complete three-dose primary series according to the standard 0-, 1-, and 6-month immunization schedule routinely used in vaccination clinics. In contrast, a single booster dose was administered to individuals who tested negative for HBsAg, HBeAg, anti-HBe, and anti-HBc but had detectable anti-HBs titers in the range of 10–100 mIU/mL. The administered hepatitis B vaccines were recombinant CHO cell-derived vaccines. Their primary antigenic component was the hepatitis B virus surface antigen, and they were adjuvanted with aluminum hydroxide. Each dose contained 20 μg of antigen in 1 mL (lot number H202310YB58), and all vaccines were manufactured by North China Pharmaceutical Jintan Biotechnology Co., Ltd. (Shijiazhuang, China). All participants completed a unified questionnaire survey.

The research team developed the questionnaire independently by reviewing relevant domestic and international literature and invited experts to evaluate and refine it. Face-to-face surveys were conducted by uniformly trained investigators. The main data collected included height, weight, age, education level, smoking history, and alcohol consumption history. To avoid recall bias, information on chronic diseases among the participants was obtained by staff from community health service stations through queries of the “Zhangjiagang City Public Health Information Platform” and telephone follow-ups prior to the administration of hepatitis B booster vaccines. A dual-entry verification method was used for questionnaire data entry to ensure accuracy and consistency.

### 2.3. Sample Collection and Testing

Venous blood samples (3–5 mL) were collected from participants who had completed the hepatitis B booster vaccination at 2–3 months post-vaccination. Blood collection was conducted in two phases: January–February and July–August 2025. Samples were centrifuged and cryopreserved by professional staff at community health centers before being promptly transported to the laboratory for quantitative anti-HBs testing. Anti-HBs titers were measured using a magnetic particle chemiluminescence immunoassay with commercial kits (manufacturer: Zhengzhou Autobio Biotechnology Co., Ltd., Zhengzhou, China, batch numbers: 240806, 250408). The anti-HBs assay demonstrated a limit of detection (LOD) of 0.5 mIU/mL and a lower limit of quantification (LLOQ) of 2 mIU/mL.

### 2.4. Statistical Analysis

Data organization was performed using WPS Office version 2022 (Beijing Kingsoft Office Software Co., Ltd., Beijing, China), and statistical analyses were conducted using IBM SPSS Statistics software version 20.0 (IBM Corp., Armonk, NY, USA). Categorical variables were described using rates or proportions, and the Chi-square test was employed for intergroup comparisons. As anti-HBs concentrations were not normally distributed, these data are presented as the median concentration (MC) and interquartile range (IQR). The Wilcoxon signed-rank test was applied to compare the groups.

Potential influencing factors for anti-HBs detection results—such as body mass index (BMI), age, education level, history of chronic disease, smoking, and drinking history, among others—were first analyzed using univariate logistic regression to calculate crude odds ratios (*ORs*) and 95% confidence intervals (*CIs*). With reference to the work of Zhang, Z et al. [22], we concur that the purposeful selection method strikes a balance between statistical significance and professional rationality. Consistent with this approach, variables demonstrating a significant association (*p* < 0.25) in univariate analysis were further included in a multivariate logistic regression model for the identification of final independent influencing factors. In this study, an optimal anamnestic response was defined as a post-booster anti-HBs titer exceeding 1000 mIU/mL, which served as the reference category for analysis. Therefore, study participants with anti-HBs titers < 1000 mIU/mL after the booster vaccination were compared with those in the reference group. We evaluated the effect of pre-booster anti-HBs concentration on the post-booster anti-HBs level using ROC (receiver operating characteristic curve) analysis.

Chinese adults were categorized by BMI as follows [23]: underweight (<18.50 kg/m^2^), normal (18.50–23.9 kg/m^2^), overweight (24.0–27.9 kg/m^2^), and obese (≥28.0 kg/m^2^). The significance level was set at 0.05, and results with *p* < 0.05 were deemed statistically significant.

## 3. Results

### 3.1. Total Participants

**Figure 1:**
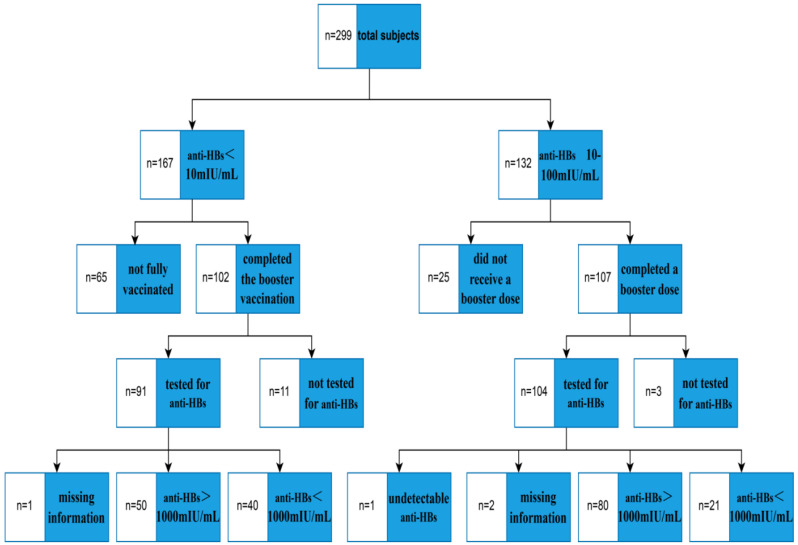
Study results flow chart.

In total, from the 167 subjects (anti-HBs < 10 mIU/mL, Cohort 1) who required a three-dose hepatitis B vaccine booster regimen, 102 completed all vaccinations. Among them, 90 participated in both blood sampling and questionnaire surveys (one was excluded due to incomplete data), yielding a follow-up success rate of 53.89%. Among the 132 subjects (anti-HBs 10–100 mIU/mL, Cohort 2) indicated for a single-dose booster, 107 received the vaccination. A total of 104 subjects underwent blood collection and questionnaire administration; however, 1 subject had unsuccessful antibody testing, and 2 provided incomplete information, resulting in 101 valid cases and a follow-up success rate of 76.52%. A total of 191 subjects (90 subjects in Cohort 1 and 101 subjects in Cohort 2) from both cohorts were included in the final analysis. Two months after completion of the hepatitis B vaccination series, anti-HBs titers were measured in the two cohorts. In Cohort 1, 50 subjects [50/(50 + 40) = 55.56%] exhibited anti-HBs concentrations greater than 1000 mIU/mL, while in Cohort 2, 80 subjects [80/(80 + 21) = 79.21%] demonstrated anti-HBs concentrations exceeding 1000 mIU/mL (Figure 1).

### 3.2. Chronic Conditions Among the Study Participants

Prior to the booster vaccination, 146 participants in this study had been diagnosed with at least one of the following chronic conditions: diabetes, hypertension, stroke, coronary heart disease, cancer, or chronic obstructive pulmonary disease (COPD). The number of cases for each condition was 95, 102, 27, 14, 14, and 1, respectively.

### 3.3. Comparison of Characteristics of Study Participants

Comparison of baseline characteristics between the two cohorts demonstrated comparable distributions for key variables, such as sex, age, and BMI, among others, with no statistically significant differences observed in any of these characteristics (Table 1).

### 3.4. Serological Testing of the Study Participants

**Figure 2:**
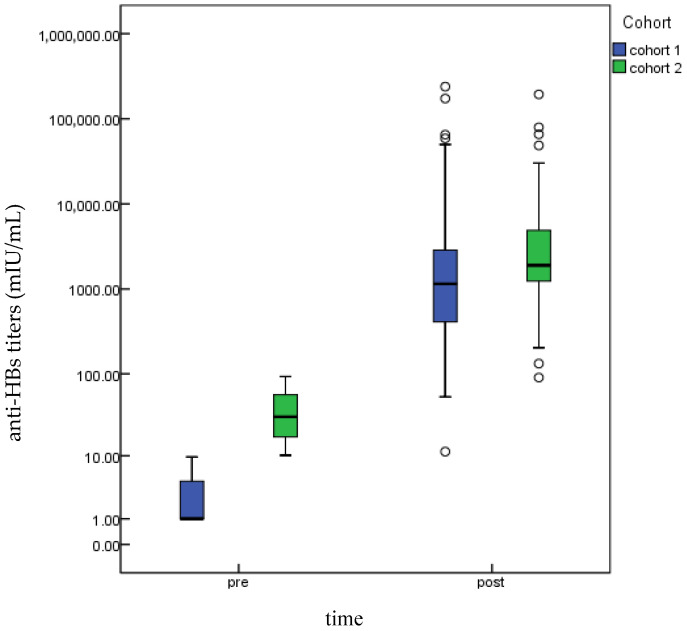
Anti-HBs titers pre- and post-booster vaccination in different cohorts. The horizontal line inside the box indicates the median; the box itself spans the IQR; the whiskers extend to the minimum and maximum values excluding outliers; and individual dots represent outliers.

Quantitative detection of hepatitis B serologic markers in the two cohorts post-blood collection revealed the following: In Cohort 1, all participants exhibited anti-HBs concentrations greater than 10 mIU/mL after booster vaccination. The post-booster anti-HBs titers (median concentration: 1149.87 mIU/mL, IQR: 408.61–2895.00 mIU/mL) were significantly higher than the pre-booster titers (median concentration: <2 mIU/mL, IQR: <2–4.56 mIU/mL), and this difference was statistically significant (*Z* = −8.24, *p* < 0.001). In Cohort 2, the post-booster anti-HBs titers (median concentration: 1897.16 mIU/mL, IQR: 1230.55–4942.77 mIU/mL) were also significantly higher than the pre-booster titers (median concentration: 30.54 mIU/mL, IQR: 17.21–56.91 mIU/mL), and the difference was statistically significant (*Z* = −8.73, *p* < 0.001). The results are shown in Figure 2.

### 3.5. Factors Associated with Hepatitis B Booster Response

Subjects with a pre-booster anti-HBs titer of 10–100 mIU/mL were less likely to exhibit a post-booster titer of <1000 mIU/mL compared to those with a pre-booster titer of <10 mIU/mL (*OR*: 0.33; 95% *CI*: 0.17–0.62). No statistically significant associations were observed between the post-booster anti-HBs titer and the other characteristics examined (*p* > 0.05). It is noteworthy that the presence of chronic diseases was not associated with achieving an anti-HBs titer > 1000 mIU/mL following booster vaccination (*p* = 0.616) (Table 2).

Multivariable logistic regression was performed to assess the association between variables with a *p*-value < 0.25 in the univariate analysis (including anti-HBs titer before booster, smoking status, and drinking history) and the anti-HBs titer following HBV booster vaccination. Compared to those with a low pre-booster titer, subjects with a high pre-booster anti-HBs titer were significantly less likely to exhibit a post-booster titer < 1000 mIU/mL (*OR* = 0.30, 95% *CI*: 0.16–0.58, *p* < 0.001). However, no other baseline characteristics showed significant associations with the post-booster anti-HBs titers (Table 3). ROC analysis was employed to assess the predictive value of the pre-booster anti-HBs concentration for a high-level immune response after the booster, with a high-level response defined as an anti-HBs titer > 1000 mIU/mL. The area under the curve (AUC) was 0.70 (95% *CI*: 0.62–0.78; *p* < 0.001). At the maximum Youden’s index, the optimal pre-booster anti-HBs cut-off concentration was determined to be 4.58 mIU/mL. Consequently, individuals with a pre-booster anti-HBs concentration ≥ 4.58 mIU/mL were more likely to achieve a post-booster anti-HBs level > 1000 mIU/mL compared to those with a concentration below this threshold.

## 4. Discussion

The anti-HBs titer is a key metric for assessing the efficacy of hepatitis B vaccination. After vaccination, the anti-HBs concentration tends to decline over time, and the reduced antibody levels may be insufficient to protect against hepatitis B virus infection, thereby posing a potential risk of infection [24,25]. However, the appropriate strategy for administering booster doses of the hepatitis B vaccine remains unclear [26]. To date, China has not established a booster immunization strategy for hepatitis B vaccination in adults. To enhance the prevention of hepatitis B virus infection, the development of a booster immunization protocol for the hepatitis B vaccine is an urgent issue requiring resolution.

This study administered booster doses to elderly individuals with insufficient anti-HBs levels who had completed the full course of hepatitis B vaccination in 2015. Participants received different numbers of vaccine doses based on their specific anti-HBs concentration intervals. Participants with anti-HBs concentrations < 10 mIU/mL showed poorer compliance with the three-dose schedule. Additionally, vaccine hesitancy was observed among some participants during follow-up. Evidence suggests that higher-dose, fewer-dose vaccination schedules may yield a relatively poorer immune response in older adults [27]. Therefore, to improve vaccination compliance, further exploration of immunization strategies remains necessary. Concurrently, efforts should also focus on refining communication strategies and methods to mitigate vaccine hesitancy. Regarding chronic conditions among the participants, the vast majority had diabetes or hypertension. Previous studies indicate that chronic disease patients may exhibit weaker responses to hepatitis B vaccination [20]^;^ however, whether their immune recall response is affected remains unknown. Therefore, incorporating chronic diseases as a factor in this study allows for an evaluation of their impact on the immune recall response.

The magnitude of the anamnestic immune response can be quantified by the serum anti-HBs titers in response to hepatitis B vaccine booster immunization [28,29]. No significant differences in baseline characteristics were observed between Cohort 1 and Cohort 2, indicating that the two cohorts were comparable. Following booster immunizations with the hepatitis B vaccine, both groups exhibited a marked increase in anti-HBs concentrations, achieving robust levels and demonstrating a potent anamnestic immune response. This study demonstrates persistent immune memory in elderly individuals nine years after hepatitis B vaccination. The observation that a subset of participants had experienced a waning of anti-HBs levels to below 2 mIU/mL following complete primary immunization in 2015 suggests that immunological memory may outlast detectable antibody titers. These findings are corroborated by other investigations involving participants from diverse age groups. A domestic study reported that subjects with anti-HBs < 10 mIU/mL 18–20 years post-vaccination exhibited rapid seroconversion after receiving booster immunization, indicating preserved anamnestic response [30]. Similarly, an international study found that most individuals maintained the capacity to mount protective immune recall upon booster administration, even 35 years after primary vaccination [26].

Univariate analysis identified no significant associations between sex, age, BMI, drinking history, or other factors and the potent anamnestic response to hepatitis B vaccine, which is consistent with previous findings [29]. These factors, though recognized as risk factors for compromised initial immunization efficacy [24], show limited effect on the anamnestic response following booster immunization, in contrast to their effect on primary anti-HBs waning. Likewise, a history of chronic diseases was not observed to affect the anamnestic response, aligning with the findings of a previous international investigation [31]. Multivariable analysis demonstrated a significant correlation between the pre-booster anti-HBs level and the magnitude of the anamnestic response, which aligns with international findings [32]. In contrast, the cohort with a pre-booster anti-HBs level < 10 mIU/mL that received three immunizations mounted a weaker response than the cohort with levels of 10–100 mIU/mL receiving one immunization. This clearly establishes the pre-booster anti-HBs level, not the number of immunizations, as the primary determinant of the recall response. The anamnestic immune response observed after booster vaccination in both cohorts supports the feasibility of a differentiated vaccination strategy, wherein individuals with different levels of antibody concentration receive distinct immunization regimens. This finding holds certain significance for optimizing the allocation of medical resources. This study found that the pre-booster concentration of anti-HBs was significantly associated with the immune recall response. According to the relevant literature [33,34], factors influencing this anamnestic response also include the frequencies of HBsAg-specific memory B cells and CD4^+^ T cells, as well as specific types of human leukocyte antigen (HLA) alleles, among others. However, these factors were not measured in this study, and the pre-booster anti-HBs concentration may correlate with them. Therefore, future research could consider more in-depth investigations at the cellular and molecular levels.

According to ROC analysis, the optimal pre-booster anti-HBs cut-off for predicting a strong anamnestic response was 4.58 mIU/mL. This value exceeds the thresholds (1.00 and 0.7 mIU/mL) established in prior investigations [31,35]. A plausible explanation for this divergence is the differing definitions of a serological response; the reference studies utilized lower post-booster benchmarks (10 or 100 mIU/mL), while our work applied a more stringent benchmark of 1000 mIU/mL. In contrast to the two aforementioned studies, the use of a post-booster reference concentration of 1000 mIU/mL reflects a strong anamnestic immune response. Higher peak antibody levels prolong the time until antibody titers fall below the 10 mIU/mL threshold, suggesting extended protective immunity and a potential decrease in the need for future booster vaccinations. Therefore, this study utilized 1000 mIU/mL as a reference level to evaluate the immune memory response, with the goal of providing evidence to optimize population-based immunization strategies and to support the development of longer-lasting, safer, and more cost-effective long-term protection. Furthermore, the higher baseline requirement in our study reasonably results in a higher pre-booster predictive cut-off, which validates the rationale behind our finding of 4.58 mIU/mL.

This study has several limitations. First, the limited sample size may limit the generalizability of our results. Second, it was not possible to adequately differentiate between an anamnestic immune response and a primary immune response following the booster vaccination. Third, the assessment of immune memory in this study relied solely on humoral immunity (antibody titers), and the potential contribution of cellular immunity was not evaluated. Fourth, the hepatitis B vaccines used in this study were based on CHO cells. This may constrain the extrapolation of the findings to vaccines that are produced with different expression systems.

## 5. Conclusions

In summary, the anamnestic immune response remains well-preserved in the elderly nine years after hepatitis B vaccination. The magnitude of this response is significantly correlated with the pre-booster anti-HBs level, while a history of chronic diseases shows no observable effects. Elderly individuals with a low immune response (anti-HBs levels of 10–100 mIU/mL after primary vaccination) demonstrated robust immune memory capacity. Given that a strong anamnestic response is observed at levels above the 4.58 mIU/mL cut-off, the focus must shift to monitoring individuals with extremely low post-vaccination anti-HBs levels to prevent breakthrough hepatitis B infections. Our findings highlight that during the implementation of hepatitis B booster vaccination, we must take into account not merely the serum antibody titer but also the existence of an immune memory recall response.

## Figures and Tables

**Table 1:** Comparison of characteristics before booster vaccination between Cohort 1 and Cohort 2.

Characteristics	Cohort 1 (Anti-HBs < 10 mIU/mL)	Cohort 2 (10 mIU/mL ≤ Anti-HBs < 100 mIU/mL)	*χ* ^2^	*p* Value
Gender, n (%)			0.26	0.609
male	43 (47.78)	52 (51.49)		
female	47 (52.22)	49 (48.51)		
Age at the time of survey, n (%)			3.24	0.198
71~	32 (35.56)	24 (23.76)		
66~70	29 (32.22)	40 (39.60)		
61~65	29 (32.22)	37 (36.63)		
BMI, n (%)			2.30	0.130
normal	33 (36.67)	48 (47.52)		
overweight or obese	57 (63.33)	53 (52.48)		
Education Level, n (%)			0.74	0.390
junior high school or above	47 (52.22)	59 (58.42)		
below junior high school	43 (47.78)	42 (41.58)		
History of Surgery, n (%)			2.03	0.154
yes	59 (65.56)	56 (55.45)		
no	31 (34.44)	45 (44.55)		
Chronic Diseases, n (%)			0.17	0.681
yes	70 (77.78)	76 (75.25)		
no	20 (22.22)	25 (24.75)		
Smoking status, n (%)			0.39	0.531
yes	18 (20.00)	24 (23.76)		
no	72 (80.00)	77 (76.24)		
Drinking history, n (%)			2.05	0.152
yes	21 (23.33)	33 (32.67)		
no	69 (76.67)	68 (67.33)		

**Table 2:** Association between post-booster anti-HBs titer and subject characteristics.

Characteristics	Post-Booster Anti-HBs <1000 mIU/mL	Post-Booster Anti-HBs >1000 mIU/mL	Univariate	
			*OR* (95% CI)	*p* Value
Gender, n (%)				
Male	32 (52.46)	63 (48.46)	1.17 (0.64, 2.16)	0.607
Female (Ref.)	29 (47.54)	67 (51.54)		
Age at the time of survey, n (%)				
71~	25 (40.98)	41 (31.54)	1.22 (0.57, 2.62)	0.611
66~70	19 (31.15)	55 (42.31)	0.69 (0.32, 1.51)	0.354
61~65 (Ref.)	17 (27.87)	34 (26.15)		
BMI, n (%)				
Normal	24 (39.34)	56 (43.08)	0.86 (0.46, 1.59)	0.626
Overweight or obese (Ref.)	37 (60.66)	74 (56.92)		
Education level, n (%)				
Junior high school or above	33 (54.10)	73 (56.15)	0.92 (0.50, 1.70)	0.790
Below junior high school (Ref.)	28 (45.90)	57 (43.85)		
History of surgery, n (%)				
Yes	34 (55.74)	81 (62.31)	0.76 (0.41, 1.41)	0.388
No (Ref.)	27 (44.26)	49 (37.69)		
Chronic diseases, n (%)				
Yes	48 (78.69)	98 (75.38)	1.21 (0.58, 2.51)	0.616
No (Ref.)	13 (21.31)	32 (24.62)		
Anti-HBs titer before booster, n (%)				
10–100 mIU/mL	21 (34.43)	80 (61.54)	0.33 (0.17, 0.62)	0.001
<10 mIU/mL(Ref.)	40 (65.57)	50 (38.46)		
Smoking status, n (%)				
Yes	18 (29.51)	24 (18.46)	1.85 (0.91, 3.75)	0.088
No (Ref.)	43 (70.49)	106 (81.54)		
Drinking history, n (%)				
Yes	21 (34.43)	33 (25.38)	1.54 (0.80, 2.98)	0.197
No (Ref.)	40 (65.57)	97 (74.62)		

**Table 3:** Multivariable logistic regression analysis of factors associated with anti-HBs levels following booster vaccination.

Post-Booster Immunization Anti-HBs Titer	Post-Booster Anti-HBs <1000 mIU/mL	
	*OR* (95% *CI*)	*p* Value
Pre-booster Anti-HBs Titer of 10–100 mIU/mL (<10 mIU/mL as Ref.)	0.30 (0.16, 0.58)	<0.001
Drinking history (non-drinkers as Ref.)	1.50 (0.70, 3.23)	0.297
Smoking status (non-smokers as Ref.)	1.75 (0.78, 3.93)	0.176

## Data Availability

The data are not publicly available due to their sensitive nature, but can be made available from the corresponding author upon reasonable request.
